# Apple Endophytic fungi and their antagonism against apple scab disease

**DOI:** 10.3389/fmicb.2022.1024001

**Published:** 2022-11-07

**Authors:** Leila Ebrahimi, Sepideh Hatami Rad, Hassan Reza Etebarian

**Affiliations:** Department of Entomology and Plant Pathology, College of Aburaihan, University of Tehran, Tehran, Iran

**Keywords:** biocontrol, chitinase activity, cellulase activity, endophyte, metabolites, volatile organic compounds

## Abstract

Endophytic fungi are microorganisms with the ability to colonize plants for the entire or at least a significant part of their life cycle asymptomatically, establishing a plant-fungus association. They play an important role in balancing ecosystems, as well as benefiting host through increasing plant growth, and protecting the host plants from abiotic and biotic stresses using various strategies. In the present study, endophytic fungi were isolated from wild and endemic apple cultivars, followed by characterizing their antifungal effect against *Venturia inaequalis*. To characterize the endophytic fungi, 417 fungal strains were separated from 210 healthy fruit, leaf, and branch samples collected from the north of Iran. Among the purified fungal isolates, 33 fungal genera were identified based on the morphological characteristics, of which 38 species were detected according to the morphological features and molecular data of ITS, *tef-1α*, and *gapdh* genomic regions (related to the genus). The results represented that most of the endophytic fungi belonged to Ascomycota (67.8%), 31.4% of isolates were mycelia sterilia, while the others were Basidiomycota (0.48%) and Mucoromycota (0.24%). Additionally, *Alternaria*, *Cladosporium*, and *Nigrospora* were determined as the dominant genera. The antifungal properties of the identified isolates were evaluated against *V. inaequalis in vitro* to determine the release of media-permeable metabolites, Volatile Organic Compounds (VOCs), chitinase, and cellulase as antifungal mechanisms, as well as producing phosphate solubilisation as growth-promoting effect. Based on the results of metabolite and VOC tests, the six isolates of *Acremonium sclerotigenum* GO13S1, *Coniochaeta endophytica* 55S2, *Fusarium lateritium* 61S2, *Aureobasidium microstictum* 7F2, *Chaetomium globosum* 2S1 and *Ch. globosum* 3 L2 were selected for greenhouse tests. Further, *Co. endophytica* 55S2 and *F. lateritium* 61S2 could solubilize inorganic phosphate. All isolates except *Ch. globosum* 3 L2 exhibited cellulase activity, while chitinase activity was observed in *Ch. globosum* 2S1, *Ch. globosum* 3 L2, and *F. lateritium* 61S2. Finally, *Co. endophytica* 55S2 and *Ch. globosum* 2S1 completely controlled the disease on the apple seedling leaves under greenhouse conditions.

## Introduction

Apple (*Malus* sp.), which belongs to the Rosaceae family, is considered as the most common and culturally important fruit crop worldwide, as well as one of the superior crops in Iran due to its nutritional and export value (Ebrahimi et al., 2016). The apple scab caused by *Venturia inaequalis* (Cooke) G. Winter is among the most main diseases in the apple-growing regions across the globe (Tenzer and Gessler, 1997), especially the areas with cool and wet spring, as well as early summer (MacHardy, 1996). The management of this disease is often based on the repeated fungicide application which is expensive and time-consuming. However, some fungicides may lose their efficacy following the development of resistance in the fungus causing apple scab. Nevertheless, the need for nonchemical control methods to reduce the crop losses is becoming increasingly important for protecting the environment and human health (Wenneker and Thomma, 2020). In this regard, a global trend tries to explore the new alternatives to synthetic fungicides, which minimize the risks associated with the development of the populations insensitive to the chemical compounds and are consistent with the food safety standards (Chen et al., 2019). Biological control is an excellent and effective alternative way to control plant diseases (Zhang et al., 2018). In addition, endophytes are a potential source of biological control agents, which are already adapted to live and persist in the plant with minimal adverse effects.

The endophytes are the microorganisms living inside plant tissues for the entire or at least a large part of their life cycle without causing any symptom or adverse harm to the host (Petrini, 1991; Saikkonen et al., 1998). The endophytic fungi can be considered as potential biological competitors since they have evolved to exploit the same resources as plant pathogens (Silva et al., 2018). They contribute to plant health by producing protective metabolites, inducing biotic and abiotic stress resistance in host plants, and improving their growth with phytohormones (Rai et al., 2014; Terhonen et al., 2019). The microorganisms can potentially protect plants from pathogenic fungi through a diverse array of modes of action such as direct inhibition *via* competition, antibiosis or mycoparasitism, and indirect inhibition by induced resistance (Latz et al., 2018). Recently, endophytic fungi stood out as the most common group of microorganisms elaborated in the laboratory to produce a variety of secondary metabolites such as alkaloids, flavonoids, terpenoids, volatile organic compounds (VOC), phenols and its derivatives, and different enzymes (Zhang et al., 2006; Strobel, 2018) with potential use in industrial application or as fungicides to control the growth of phytopathogenic fungi (Meenavalli et al., 2011; Toghueo et al., 2017). These microorganisms inside the plant are able to degrade a portion of plant lignin and cellulose (Li et al., 2021) which helps host plants to protect themselves against invasive pathogens (Marques et al., 2018). Also, endophytes with the ability to secrete extracellular chitinase would decompose chitin, a β-(1,4)-linked polymer of N-acetyl-D-glucosamine, and the cell wall structure of most phytopathogenic fungi along with other versatile bioactive compounds (Hartl et al., 2012). Endophytes contribute to several processes related to plant growth and development similar to the rhizospheric microbes, such as, nitrogen fixation, phosphate solubilization, etc. (Santoyo et al., 2016). Inorganic phosphate solubilization, through microorganisms, is one of the major mechanisms involved in plant growth (Adhikari and Pandey, 2019). Solubilization of inorganic insoluble phosphate salts by different microorganisms depends on their ability to produce organic acids in the respective environment. These organic acids decrease the pH of the soil or any medium, providing the facility to exchange the metal part of insoluble phosphates to potassium or sodium, resulting in the formation of soluble phosphate salts (Singh and Satyanarayana, 2011; Rinu et al., 2012) which is absorbable for plants.

Several studies have assessed the endophytic fungal communities of apple (e.g., Camatti-Sartori et al., 2005; Alijani et al., 2016a; Liu et al., 2017; Muresan, 2017; Afandhi et al., 2018; Liu et al., 2018; Arrigoni et al., 2020; Olivieri et al., 2021), some of which have focused on the biological control potential of the endophytes against apple fungal diseases. Based on the results, apple tissues are colonized by a diverse array of fungal taxa (e.g., *Alternaria* Nees, *Arthrinium* Kunze, *Aspergillus* P. Micheli ex Haller, *Biscogniauxia* Kuntze, *Botryosphaeria* Ces. & De Not., *Chaetomium* Kunze, *Colletotrichum* Corda, *Dicyma* Boulanger, *Doratomyces* Corda, *Epicoccum* Link, *Neosetophoma* Gruyter, Aveskamp & Verkley, *Paraconiothyrium* Verkley, *Penicillium* Link, *Stemphylium* Wallr., *Trichoderma* Pers., *Trichothecium* Link, *Xylaria* Hill ex Schrank, *Sporobolomyces* Kluyver & C.B. Niel, *Rhodotorula* F.C. Harrison, *Debaryomyces* Klöcker and *Cryptococcus* Kütz and etc). Alijani et al. (2016a) purified about 350 isolates from the shoots, leaves, and barks of endemic and commercial apple trees in West Azerbaijan province, Iran and identified 24 species belonging to 10 genera of Ascomycota. They examined the antagonistic properties of the isolates against *Diplodia bulgarica* A.J.L. Phillips, J. Lopes & Bobev, the causal agent of apple canker disease, *in vitro* and found various interactions in the paired combinations of endophytic fungi and *D. bulgarica* (Alijani et al., 2016b). Liu et al. (2017) highlighted the biological control potential of 81 endophytic fungi from apple shoots to preserve apple trees against *Neonectria ditissima* (Tul. & C. Tul.) Samuels & Rossman infection *in vitro*. They mentioned that 18 fungal isolates inhibited the radial growth of three *N. ditissima* isolates, among which 15 selected ones were identified as *Epicoccum*, *Chaetomium*, *Biscogniauxia*, *Neosetophoma*, and *Penicillium* species. In another study, a collection of 60 endophytic fungal isolates was obtained from apple trees in Canada, 60% of which were *Penicillium* or *Trichoderma* species (Muresan, 2017). Furthermore, 55 isolates significantly inhibited *V. inaequalis* growth *in vitro*, the most effective one of which was *Fusarium oxysporum* Schltdl. isolate FRS09 with 83% inhibition.

The endophytic fungi play roles in protecting the plant from herbivorous insects and diseases, as well as supporting the absorption process of the nutrients required for photosynthesis (Gimenez et al., 2007; Rozpądek et al., 2015; Vergara et al., 2017; Li et al., 2018). Given the crucial role of endophytic fungi, the present study focused on the endophytic fungi from wild and endemic apple cultivars in the north of Iran. In the study, the endophytic fungi were isolated from the healthy leaves, fruits, and branches of the cultivars, and tested for their efficacy against *V. inaequalis* and ability to prevent apple scab infection and its symptoms. The fungi were identified with consistent biological control capacity against apple scab *in vitro* and greenhouse tests. The results provide evidence that the naturally-occurring endophytic fungi can be a novel source for biological control agents.

## Materials and methods

### Sampling and endophytic fungi isolation

The healthy leaf, fruit, and branch samples of wild and endemic apple cultivars were collected from 70 trees in the north of Iran (Guilan, Mazandaran and Golestan provinces) during July–September 2019. They were placed in paper bags and stored at 4°C. Additionally, plant materials were disinfected based on the method modified by Strobel and Daisy (2003) (Ebrahimi et al., 2021). The fungi were separated on the three media of Water Agar (WA), Corn Meal Agar (CMA), and Potato Dextrose Agar (PDA), and hyphal tip method was applied for purification. All of the identified isolates were deposited in the Fungal Culture Collection (IRAN) of the Iranian Research Institute of Plant Protection (Tehran, Iran).

### Endophytic fungi identification

The appearance of colony, structure and color of mycelium, type of teleomorph and/or anamorph, and morphology of ascoma, conidiomata, conidia, and conidiophores (e.g., size, color, shape, and ornamentation), as well as conidiogenous cells, and spore production mechanism were studied for examining fungi morphologically (Ellis, 1971, 1976; Sutton, 1980; Sivanesan, 1987; Klich and Pitt, 1988; Klich, 2002; Leslie and Summerell, 2006; Simmons, 2007). Each of the fungal isolates was separately sub-cultured on the PDA; Czapek Yeast Extract Agar (CYA; sucrose 30 g, yeast extract 5 g, K_2_HPO_4_ 1 g, NaNO_3_ 3 g, KCl 0.05 g, MgSO_4_.7H_2_O 0.05 g, FeSO_4_.7H_2_O 1 mg, agar 20 g/l, final pH 6.0–6.5); Carnation Leaf Agar (CLA; autoclaved Carnation leaves pieces on nearly solid 2% WA; agar 20 g/l water); Corn Meal Agar (CMA; corn meal infusion from 50 g solids, agar 20 g/l); Oatmeal Agar (OA; oat meal infusion from 30 g solids, agar 20 g/l); Potato Carrot Agar (PCA; potato infusion from 20 g solids, carrot infusion from 20 g solids, agar 20 g/l); Malt Extract Agar (MEA; malt extract 30 g, peptone 5 g, agar 20 g/l); Synthetic Nutrient Agar (SNA; KH_2_PO_4_ 1 g, KNO_3_ 1 g, MgSO_4_.7H_2_O 0.5 g, KCl 0.5 g, Glucose 0.2 g, Sucrose 0.2 g, agar 20 g/l); and Tap Water Agar plus wheat straw (TWA; agar 20 g/l tap water plus autoclaved wheat straw) for inducing sporulation. Further, microscopic slides were prepared in lacto-phenol or lacto-phenol cotton blue solutions after 7, 14, and/or 30 days (due to the fungal species), followed by assessment under a BH2 light microscope (Olympus, Japan). The isolates were classified into morphotypes according to their morphological appearance, and at least one isolate of each morphotype was kept for molecular identification.

Furthermore, the rapid simplified DNA extraction protocol provided by Cenis (1992) was employed for the DNA extraction from seven-day-old fresh mycelia. The fungal isolates were molecularly identified based on the Internal Transcribed Spacer (ITS)-rDNA, glyceraldehyde-3-phosphate dehydrogenase (*gapdh*), and translation elongation factor 1-alpha (*tef-1α*) sequences which were, respectively, amplified by using the ITS1/ITS4 (White et al., 1990), EF1/EF2 (O'Donnell et al., 1998) and gpd1/gpd2 (Berbee et al., 1999) primer pairs related to the fungal genus. In the present study, the reaction mixture and PCR conditions for ITS, *tef-1α*, and *gapdh* were the same as those presented by Ebrahimi and Fotouhifar (2016), O'Donnell et al. (1998), and Song et al. (2019), respectively. PCR products were purified and directly sequenced in one direction with ITS1, EF1, and gpd1 primers by BGI Company (Denmark), respectively.

The ITS, *tef-1α*, and *gapdh* sequences were compared with those of the most closely-related fungal species according to the NCBI BLAST program, relevant websites, observed colony, and spore morphology to confirm the taxonomic status of the intended fungal isolates. Finally, the sequence data were deposited in the GenBank database.

For phylogenetic analyses, sequences of genomic regions of *gapdh*, *tef-1α*, and ITS from different species were aligned with the homologous reference sequences of the respective genomic regions of related species obtained from GenBank (Supplementary Table S1) using ClustalW (Thompson et al., 1994). Maximum likelihood (ML) (Felsenstein, 1981) analysis was done by heuristic search with MEGA software ver. 7 (Kumar et al., 2016). Models TN93 + G, K2 + G and K2 + I were recommended by MEGA as the optimal nucleotide substitution models for *gapdh*, *tef-1α*, and ITS data, respectively. Characters were treated as un-weighted and unordered with gaps treated as missing data. Confidence of individual clades was assessed by ML bootstrap analysis (Felsenstein, 1985) with 1,000 replicates.

### Biocontrol experiments *in vitro*

#### Cellophane membrane-based method

The cellophane membrane-based method (Dennis and Webster, 1971) was performed in duplicate to screen the endophytic isolates based on their antagonism against *V. inaequalis* (IRAN 16870 F). Then, the isolates expressing the visually-detected antifungal activity (Table 1) were subjected to a secondary screening (the cellophane membrane-based method in triplicate). In this technique, a sterile cellophane membrane of the same diameter as a Petri plate was overlaid on the PDA medium by using sterile forceps. A disc of endophytic isolate was inoculated at the membrane center and maintained at 20°C for 3–5 days related to isolate growth speed. Following incubation, the isolate culture along with the membrane was carefully removed from the plate, and a plug of *V. inaequalis* was positioned on the plate and kept at 20°C for 1 month. The colony diameter of *V. inaequalis* was measured and compared to the value obtained by culturing *V. inaequalis* on fresh PDA plates (control treatment). The percentage of growth inhibition was calculated by using the formula n = (a − b)/a × 100, where n is considered as the growth inhibition percentage, a indicates the colony diameter of uninhibited *V. inaequalis*, and b shows the colony diameter of antagonist-treated *V. inaequalis* (Etebarian et al., 2005).

**Table 1:** Identified endophytic fungi of Iranian endemic and wild apple based on morphology and by ITS, translation elongation factor 1-alpha (*tef-1α*) and glyceraldehyde-3-phosphate dehydrogenase (*gapdh*) sequences.

Isolate	Species	Host	Geographical region	Collection accession number	Genbank accession number
					**ITS**
2S1	*Chaetomium globosum*	Endemic apple branch	Golestan, Gonbad Kavous	IRAN 4355C	MZ151358
3 L2	*Chaetomium globosum*	Endemic apple leaf	Golesta, Minoudasht	IRAN 4356C	MZ151359
7F2	*Aureobasidium microstictum*	Wild apple fruit	Golesta, Minoudasht	IRAN 4378C	MZ151360
11S1	*Venturia inaequalis*	Wild apple branch	Golestan, Ramian	IRAN 4379C	MZ151361
12S2	*Penicillium chrysogenum*	Wild apple branch	Golestan, Ramian	IRAN 4357C	MZ151362
16 L1	*Alternaria infectoria*	Wild apple leaf	Golestan, Ramian	IRAN 4380C	MZ151363
17S2	*Aposphaeria corallinolutea*	Endemic apple branch	Golestan, Khanbebin	IRAN 4381C	MZ151364
GO2L3	*Hypoxylon fragiforme*	Endemic apple leaf	Golesta, Minoudasht	IRAN 4382C	MZ151387
GO2S2	*Neosetophoma salicis*	Endemic apple branch	Golesta, Minoudasht	IRAN 4383C	MZ151388
GO3S1	*Neosetophoma salicis*	Endemic apple branch	Golesta, Minoudasht	IRAN 4384C	MZ151389
GO5L3	*Nigrospora oryzae*	Endemic apple leaf	Golestan, Gonbad Kavous	IRAN 4358C	MZ151390
GO5S1	*Talaromyces verruculosus*	Endemic apple branch	Golestan, Gonbad Kavous	IRAN 4359C	MZ151391
GO7L1	*Nemania serpens*	Endemic apple leaf	Golesta, Minoudasht	IRAN 4385C	MZ151392
GO8S2	*Hydeomyces desertipleosporoides*	Endemic apple branch	Golesta, Minoudasht	IRAN 4386C	MZ151393
GO13S1	*Acremonium sclerotigenum*	Endemic apple branch	Golestan, Ramian	IRAN 4360C	MZ151394
22S4	*Discostroma corticola*	Endemic apple branch	Mazandarn, Kiasar	IRAN 4387C	MZ151365
23 L3	*Aspergillus terreus*	Endemic apple leaf	Mazandarn, Kiasar	IRAN 4361C	MZ151366
32 L2	*Ramularia* sp.	Wild apple leaf	Mazandarn, Neka	IRAN 4388C	MZ151367
33 L1	*Alternaria tenuissima*	Endemic apple leaf	Mazandarn, Neka	IRAN 4362C	MZ151368
33 L6	*Stachybotrys chartarum*	Endemic apple leaf	Mazandarn, Neka	IRAN 4397C	OM674386
36F4	*Neoscytalidium dimidiatum*	Endemic apple fruit	Mazandarn, Polsefid	IRAN 4363C	MZ151369
39S5	*Gibellulopsis nigrescens*	Endemic apple branch	Mazandarn, Savadkouh	IRAN 4389C	MZ151370
39S6	*Chaetomium globosum*	Endemic apple branch	Mazandarn, Savadkouh	IRAN 4364C	MZ151371
43 L3	*Pseudoanthostomella sepelibilis*	Endemic apple leaf	Guilan, Siahkal	IRAN 4390C	MZ151372
44 L1	*Nigrospora oryzae*	Endemic apple leaf	Guilan, Paresar	IRAN 4398C	MZ151373
47 L1	*Colletotrichum godetiae*	Wild apple leaf	Guilan, Paresar	IRAN 4391C	MZ151375
47F1	*Aspergillus versicolor*	Wild apple fruit	Guilan, Paresar	IRAN 4365C	MZ151374
51 L3	*Nigrospora oryzae*	Endemic apple leaf	Guilan, Rezvanshahr	IRAN 4399C	MZ151376
52 L2	*Coprinopsis atramentaria*	Endemic apple leaf	Guilan, Fouman	IRAN 4392C	MZ151377
53 L1	*Colletotrichum gloeosporioides*	Endemic apple leaf	Guilan, Fouman	UT53 L1	MZ151386
54 L1	*Annulohypoxylon stygium*	Endemic apple leaf	Guilan, Siahkal	IRAN 4393C	MZ151378
55S2	*Coniochaeta endophytica*	Endemic apple branch	Guilan, Paresar	IRAN 4366C	MZ151379
56F3	*Gleoephyllum trabeum*	Endemic apple fruit	Guilan, Bazarjomee	IRAN 4367C	MZ151380
59 L6	*Pestalotiopsis lespedezae*	Wild apple leaf	Guilan, Paresar	IRAN 4396C	MZ151381
60S1	*Paecillomyces maximus*	Wild apple branch	Guilan, Fouman	IRAN 4368C	MZ151383
62 L1	*Nigrospora oryzae*	Endemic apple leaf	Guilan, Rezvanshahr	IRAN 4394C	MZ151384
62 L3	*Neopestalotiopsis clavispora*	Endemic apple leaf	Guilan, Rezvanshahr	IRAN 4369C	MZ151385
60 L4	*Colletotrichum fructicola*	Wild apple leaf	Guilan, Fouman	IRAN 4395C	MZ151382
					*gapdh*
58 L2	*Curvularia hominis*	Wild apple leaf	Guilan, Siahkal	IRAN 4400C	MZ339272
26S2	*Curvularia spicifera*	Endemic apple branch	Mazandarn, Kiasar	IRAN 4370C	MZ339270
33S2	*Curvularia spicifera*	Endemic apple branch	Mazandarn, Neka	IRAN 4371C	MZ339271
					*tef-1α*
61S2	*Fusarium lateritium*	Endemic apple branch	Guilan, Siahkal	IRAN 4470C	MZ339268
25S3	*Fusarium incarnatum*	Endemic apple branch	Mazandarn, Kiasar	IRAN 4372C	MZ339266
37F6	*Fusarium fujikuroi*	Endemic apple fruit	Mazandarn, Polsefid	IRAN 4373C	MZ339267
GO2L1	*Fusarium acuminatum*	Endemic apple leaf	Golesta, Minoudasht	IRAN 4374C	MZ339269

#### Volatile organic compound-mediated interactions

In addition, the effect of endophyte Volatile Organic Compounds (VOCs) on *V. inaequalis* was examined by using sandwiched Petri plates explained by Lillbro (2005). After inoculating the endophytic isolates and *V. inaequalis* on the PDA plates, *V. inaequalis* plates were placed on top of an isolate plate, sealed with Parafilm, and incubated at 20°C. Further, plates with *V. inaequalis* were sandwiched with uninoculated PDA plates as a control treatment. Each treatment was repeated three times. The colony diameter of *V. inaequalis* was obtained after 1 month and the above-mentioned formula was used to compute the growth inhibition percentage.

#### Chitinase activity

The method of Hsu and Lockwood (1975) was applied to determine chitinase production. Briefly, the endophytic isolates were grown on the chitin agar containing 0.4% colloidal chitin and 1.5% agar adjusted to pH 7.2. Furthermore, the colloidal chitin was prepared according to Berger and Reynolds (1958). The plates were incubated for 5 days at 25°C. The ability of chitinase production was revealed by a clear halo around the colonies. Ultimately, the ratio of the clear zone diameter to colony diameter was calculated as chitinase activity.

#### Cellulase activity

Regarding congo red cellulase activity, all fungal isolates were developed on the carboxymethyl cellulose (CMC) agar medium consisting of 0.4 g/l KH_2_PO_4_, 0.02 g/l CaCl_2_, 0.02 g/l NaCl, 0.02 g/l FeSO_4_ 7H_2_O, 2.5 g/l CMC, and 15.0 g/l agar at 25°C for 7 days. The pH was set to 7.2 by using 1 M NaOH. To visualize the hydrolysis zone, agar medium was flooded with an aqueous congo red solution (1 mg/ml) for 20 min. Then, congo red solution was poured off, and the plates were further treated by flooding with 1 M NaCl for 15 min. The cellulase activity was obtained by measuring the diameter of the clear zone around each colony. In fact, it was computed as the ratio of the clear zone diameter to colony diameter (Majidi et al., 2011).

#### Phosphate solubilization

To assess phosphate solubilization ability, a five-mm agar plug of the endophytic isolate was placed in the center of a plate containing the Sperber medium (Sperber, 1958) prepared with insoluble phosphate. Before autoclaving, the medium pH was adjusted to 7.2. The cultures were incubated at 25°C for 7 days. Then, solubilization index was evaluated based on the ratio of the clear zone diameter to colony diameter.

#### Greenhouse experiments

Additionally, the two-year-old seedlings of Golab apple cultivar., which is sensitive to apple scab disease, were utilized in this study. The pathogenicity test was first conducted to ensure the pathogenicity of *V. inaequalis* isolate and optimize the greenhouse conditions for further tests. The conidia suspension (10^5^ conidia/ml) of *V. inaequalis* was sprayed on the apple leaves. Further, all of the inoculated branches were individually covered with plastic bags for maintaining a relative humidity of 100% for 48 h. Then, the pots were kept in a greenhouse with the humidity of >80% at 18–20°C, followed by the daily monitoring of the symptom appearance.

The selected endophytic isolates were tested for biological control potential. For this purpose, the apple seedlings were inoculated with endophytic isolates at a final concentration of 10^8^ conidia/ml. At 48 h after antagonist inoculation, *V. inaequalis* suspension was inoculated. To do this, as we needed much amount of pathogen suspension, hyphal suspension of the pathogen was prepared from 3-week-old colony grown on PDA (in 6 cm plates) at 25°C and homogenized in distilled water. The culture of the isolate was put into a mortar and ground with a small amount of sterile water to make a syrupy suspension, and it was sprayed on the apple leaves. Each branch was covered with a plastic bag for 48 h and then kept in a greenhouse with the humidity of >80% at 18–20°C. Three seedlings were sprayed with *V. inaequalis* and sterile distilled water as the positive and negative controls, respectively. Each treatment was performed on three seedlings, the tests were repeated twice, and the symptom appearance was examined daily. The infection percentage was determined by measuring individual leaf area and disease symptoms after 1 month of pathogen inoculation. Finally, the disease inhibition rate of each antagonist was calculated compared to the control. To meet Koch’s postulates, the pathogen isolate was reidentified morphologically based on structures produced on leaf spots.

#### Statistical analysis

The experiments were conducted in a completely randomized design and complete randomized block design for *in vitro* and *in vivo* tests, respectively. Data were analyzed by using SAS software version 9.0. All the data were subjected to the analysis of variance (ANOVA) followed by Duncan’s multiple range test for determining mean differences (Steel and Torrie, 1980).

## Results

### Endophytic fungal isolates

**Figure 1:**
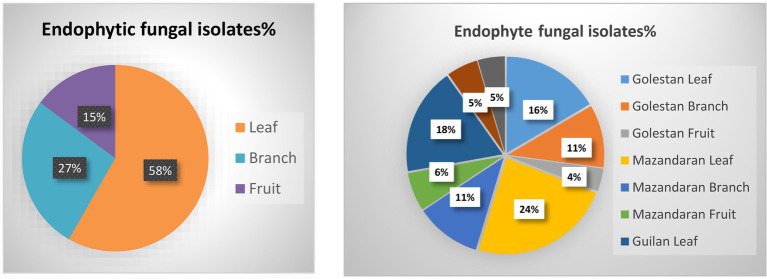
Endophytic fungal isolates were obtained from leaf, branch and fruit samples of wild and endemic apple cultivars collected from Golestan, Mazandaran and Guilan provinces of Iran.

**Figure 2:**
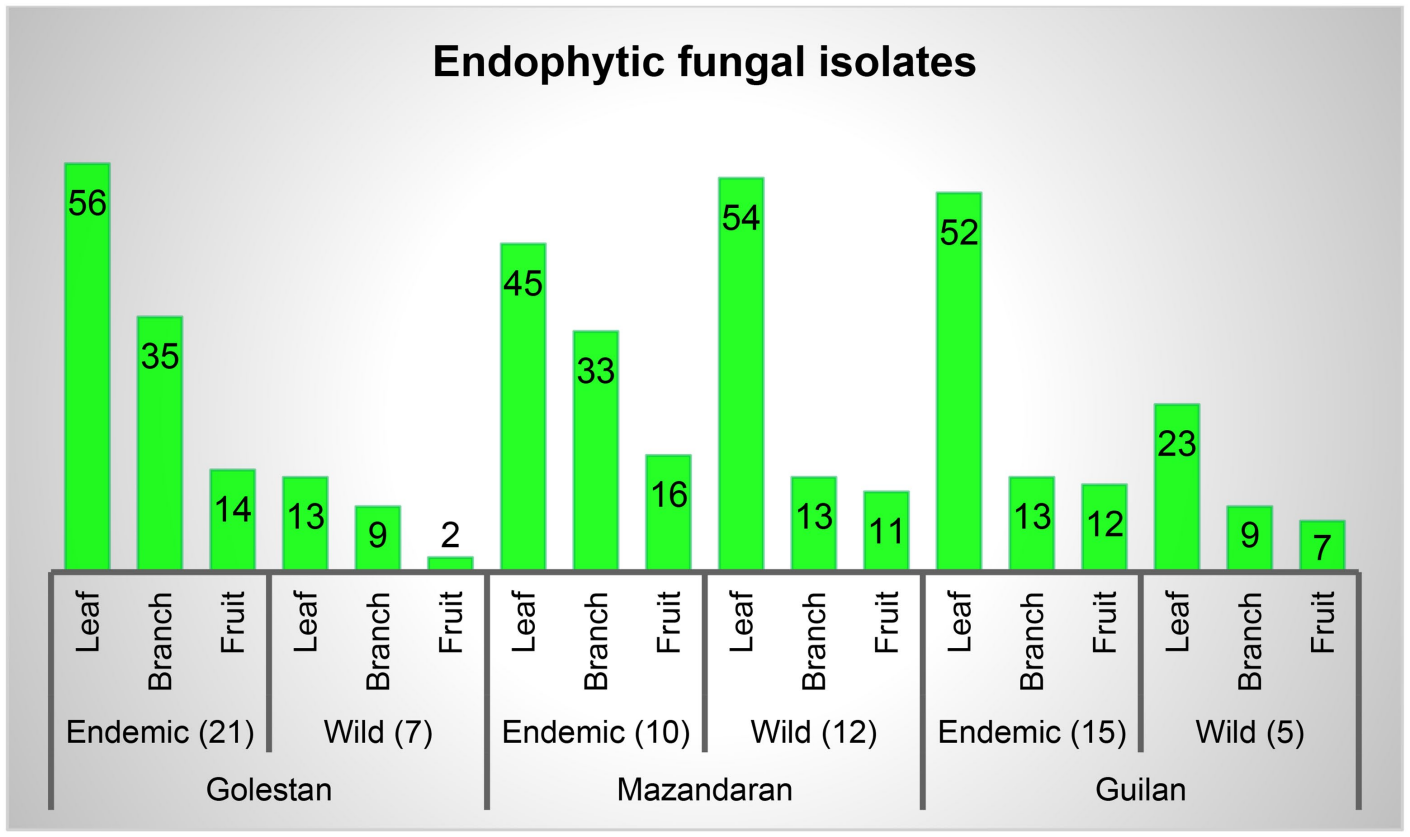
Endophytic fungal isolates percentage in leaf, branch and fruit samples, and separately in each province.

A total of 70 apple samples (each consisted of branch, leaf, and fruit) were collected, of which 28 (21 endemic and 7 wild apple), 22 (10 endemic and 12 wild apple), and 20 (15 endemic and 5 wild apple) were gathered from Golestan, Mazandaran, and Guilan provinces, respectively (Figure 1). In addition, 417 endophytic fungal isolates were obtained, among which 129 (16% leaf, 11% branch, 4% fruit), 172 (24% leaf, 11% branch, 6% fruit), and 116 (18% leaf, 5% branch, 5% fruit) were, respectively, related to Golestan, Mazandaran, and Guilan provinces (Figures 1, 2).

**Figure 3:**
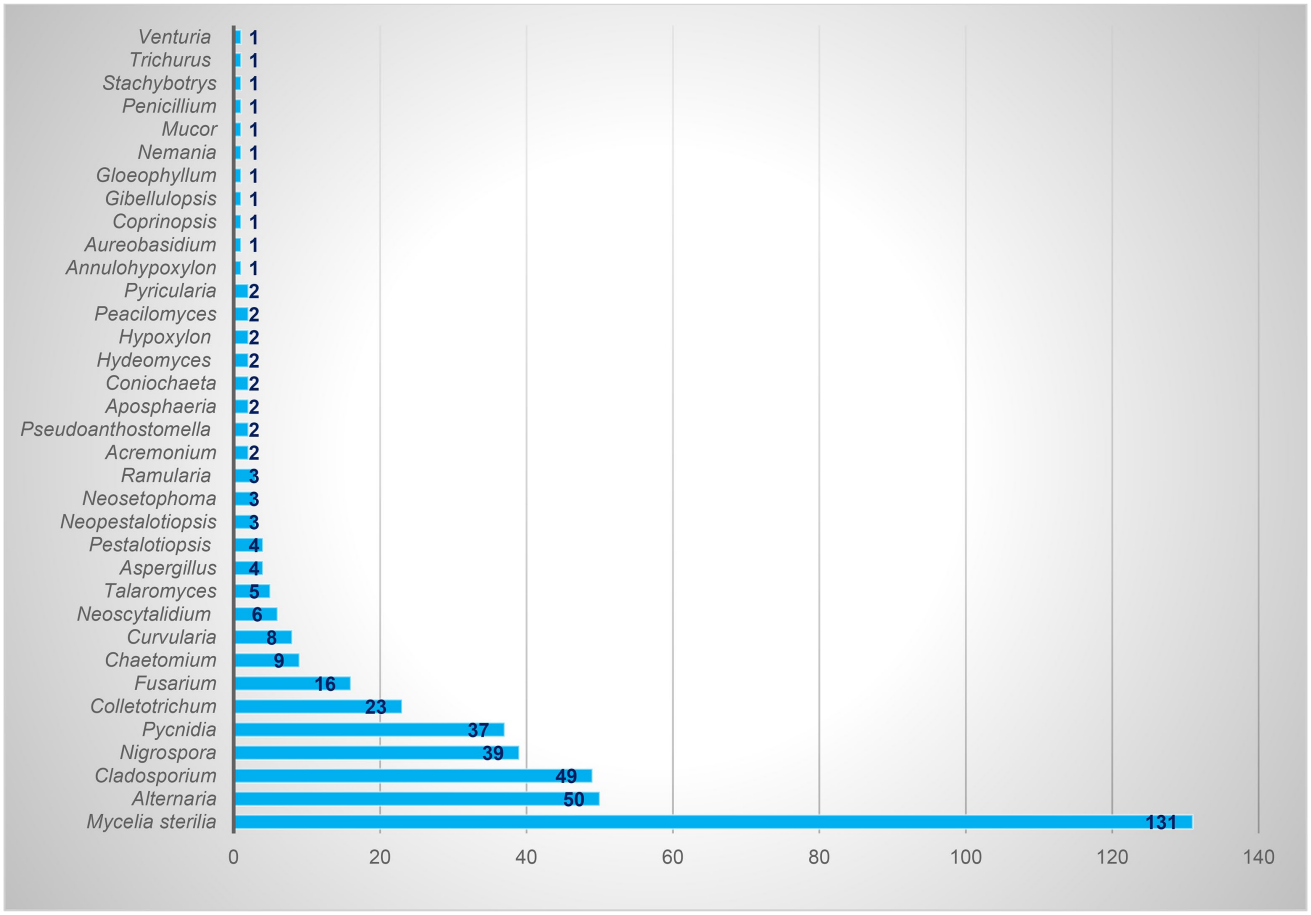
Total isolates of each identified endophytic genus.

**Figure 4:**
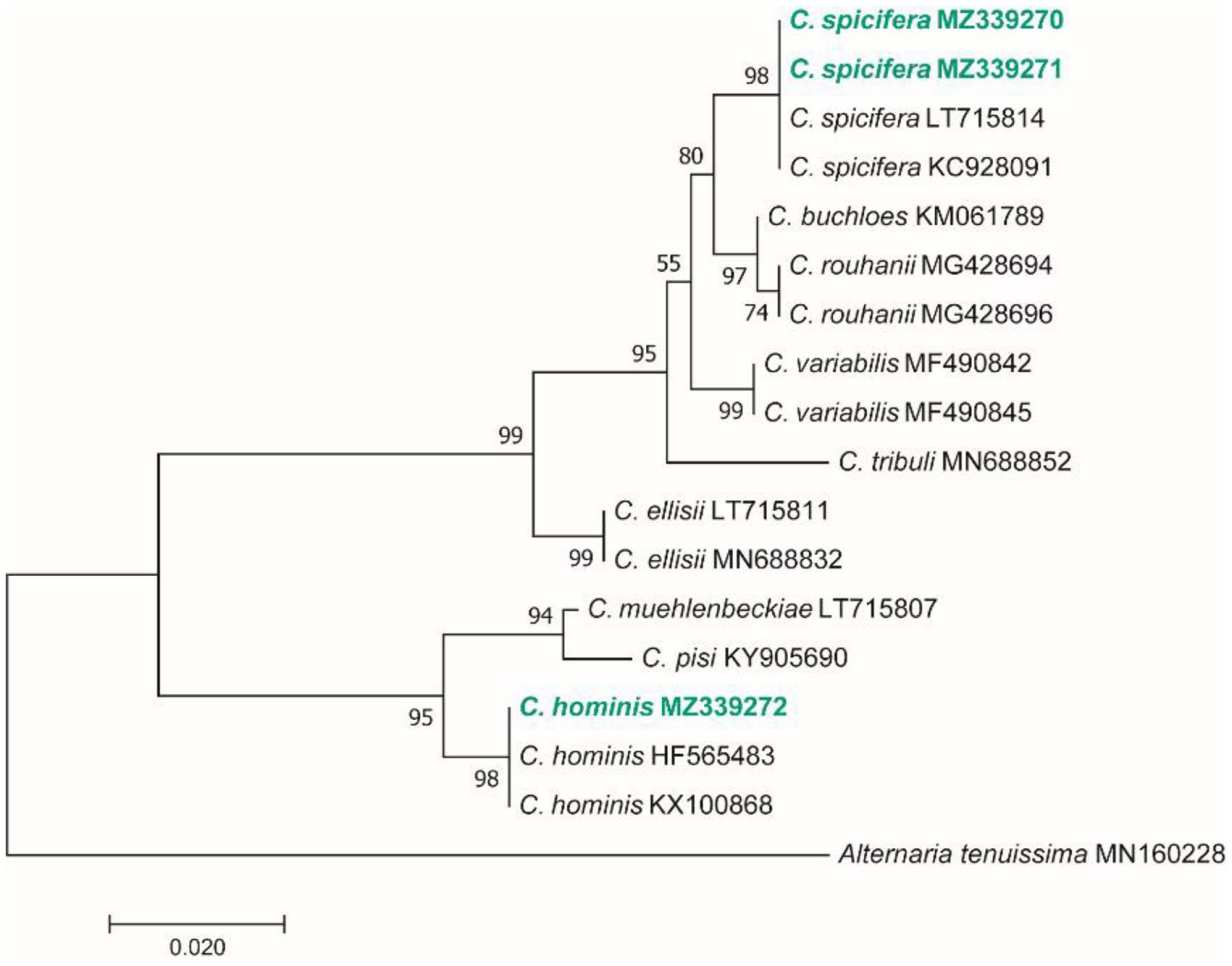
Maximum Likelihood (ML) tree based on aligned sequences of *gapdh* gene of 18 isolates generated in MEGA 7 under TN93 + G model. The tree was rooted to *Alternaria tenuissima* (IRAN 2428C). Bootstrap values (1,000 replicates) indicated at the nodes. The scale bar indicates nucleotide substitution in ML analysis, values ≥50% are shown above/below the branches. The surveyed isolates in the current study are highlighted in bold.

**Figure 5:**
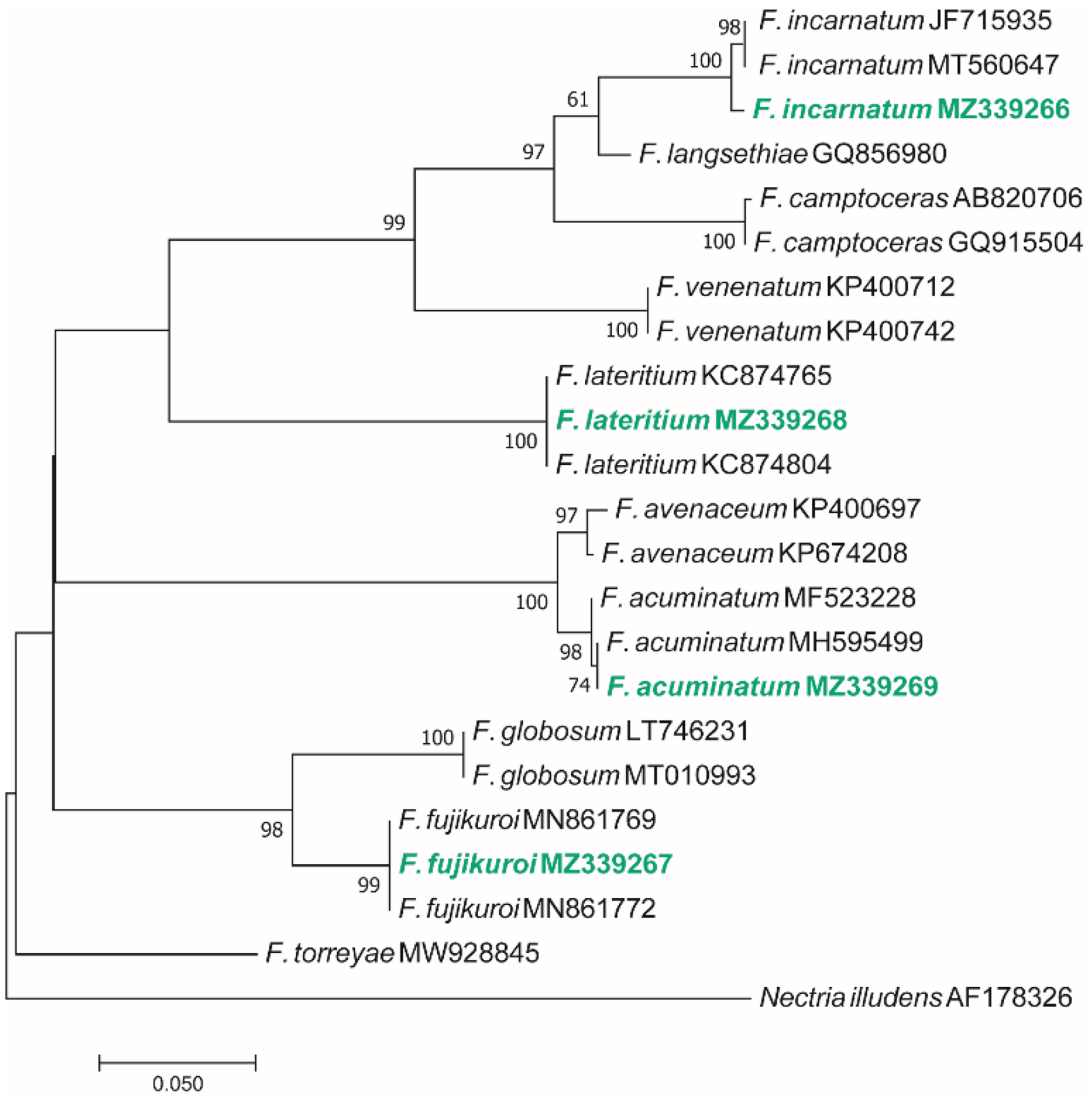
Maximum Likelihood (ML) tree based on aligned sequences of *tef-1α* gene of 23 isolates generated in MEGA 7 under K2 + I model. The tree was rooted to *Nectria illudens* (NRRL 22090). Bootstrap values (1,000 replicates) indicated at the nodes. The scale bar indicates nucleotide substitution in ML analysis, values ≥50% are shown above/below the branches. The surveyed isolates in the current study are highlighted in bold.

**Figure 6:**
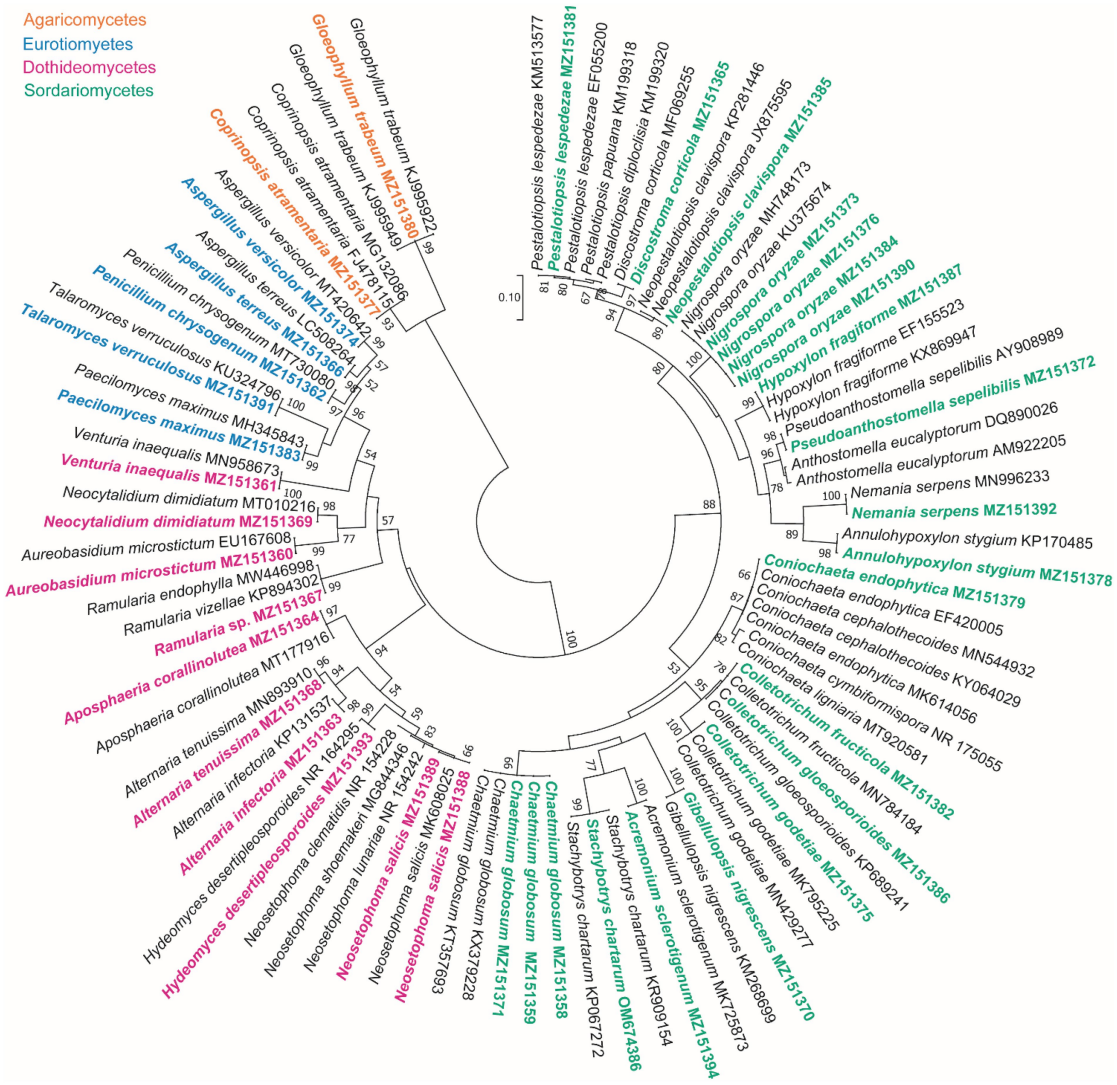
Maximum Likelihood (ML) tree based on aligned sequences of ITS region of 92 isolates generated in MEGA 7 under K2 + G model. Bootstrap values (1,000 replicates) indicated at the nodes. The scale bar indicates nucleotide substitution in ML analysis, values ≥50% are shown above/below the branches. The surveyed isolates in the current study are highlighted in bold.

Based on the morphological characteristics, 33 fungal genera were detected in the purified fungal isolates (Figure 3). Among these genera, 38 species were identified by considering the morphological properties and molecular data of *gapdh* (Figure 4), *tef-1α* (Figure 5), and ITS (Figure 6) genomic regions (Table 1). The results indicated the assignment of the detected isolates to Ascomycota (67.8%), mycelia sterilia (31.4%), Basidiomycota (0.48%), and Mucoromycota (0.24%). The species of *Coprinopsis atramentaria* (Bull.) Redhead, Vilgalys & Moncalvo, and *Gloeophyllum trabeum* (Pers.) Murrill, N. Amer. Fl. belonged to orders Agaricales and Gloeophyllales from the class Agaricomycetes of Basidiomycota, respectively. The identified species of Ascomycota were categorized into the three classes of Sordariomycetes, Dothideomycetes, and Eurotiomyetes (Figure 6). Further, the Sordariomycetes included seven separate groups related to orders Amphisphaeriales, Xylariales, Hypocreales, Glomerellales, Coniochaetales, Sordariales, as well as another group concerning *Nigrospora* Zimm. genus which is placed in Apiosporaceae, Sordariomycetidae families incertae sedis. The members of Dothideomycetes were grouped into the five orders of Venturiales, Pleosporales, Capnodiales, Dothideales, and Botryosphaeriales. The third class, Eurotiomyetes, involved the members of order Eurotiales.

### Screening of endophytic fungi for antifungal activity

In this study, the endophytic fungal isolates were evaluated for antifungal activity through employing the cellophane membrane-based method. They were tested in terms of the ability to produce antifungal media-permeable metabolites against *V. inaequalis*. The other approaches such as dual culture assay are not applicable since *V. inaequalis* is a slow-growing fungus. Furthermore, the isolates belonged to the geneus *Alternaria*, *Aspergillus*, *Colletotrichum*, *Neoscytalidium* Crous & Slippers, *Nigrospora*, *Paecilomyces* Bainier, *Pestalotiopsis* Steyaert, *Penicillium* and *Talaromyces* Visagie, N. Yilmaz & K. Jacobs did not exhibit antagonistic effect. The antifungal properties against apple scab agent were observed in 29 isolates (Table 2).

**Table 2:** Antifungal activity of endophytic fungi isolated from apple based on the cellophane membrane-based method.

Endophytic fungi	Antifungal activity
*Acremonium sclerotigenum*	+*
*Alternaria infectoria*	−
*Alternaria tenuissima*	−
*Alternaria* spp.	−
*Annulohypoxylon stygium*	±
*Aposphaeria corallinolutea*	±
*Aspergillus terreus*	−
*Aspergillus versicolor*	−
*Aureobasidium microstictum*	±
*Chaetomium globosum*	+
*Colletotrichum fructicola*	−
*Colletotrichum gloeosporioides*	−
*Colletotrichum godetiae*	−
*Colletotrichum* spp.	−
*Coniochaeta endophytica*	+
*Coprinopsis atramentaria*	±
*Curvularia hominis*	+
*Curvularia spicifera*	+
*Discostroma corticola*	+
*Fusarium acuminatum*	+
*Fusarium fujikuroi*	±
*Fusarium incarnatum*	±
*Fusarium lateritium*	+
*Gibellulopsis nigrescens*	+
*Gloeophyllum trabeum*	+
*Hydeomyces desertipleosporoides*	+
*Hypoxylon fragiforme*	+
*Nemania serpens*	+
*Neopestalotiopsis clavispora*	±
*Neoscytalidium dimidiatum*	−
*Neosetophoma salicis*	+
*Nigrospora oryzae*	−
*Paecilomyces maximus*	−
*Penicillium chrysogenum*	−
*Pestalotiopsis lespedezae*	±
*Pseudoanthostomella sepelibilis*	±
*Ramularia* sp.	+
*Stachybotrys chartarum*	+
*Talaromyces verruculosus*	−

*– Not active; ± slightly active; + active.

**Figure 7:**
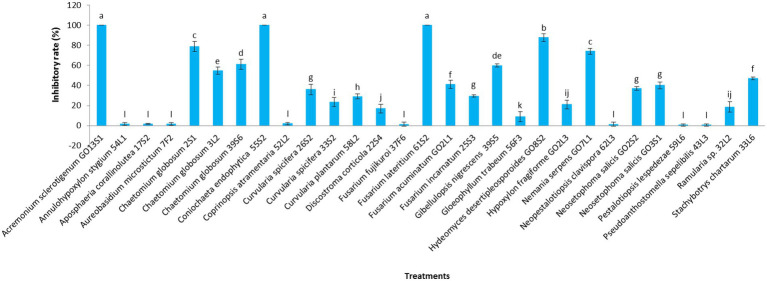
Effect of media-permeable metabolites of endophytic fungi isolated from apple on *Venturia inaequalis* mycelia growth after 1 month *in vitro*. Data are the means ± SE of three replicates. Values of histograms with common letters are not significantly different at *p* ≤ 0.01 according to Duncan’s multiple range test.

**Figure 8:**
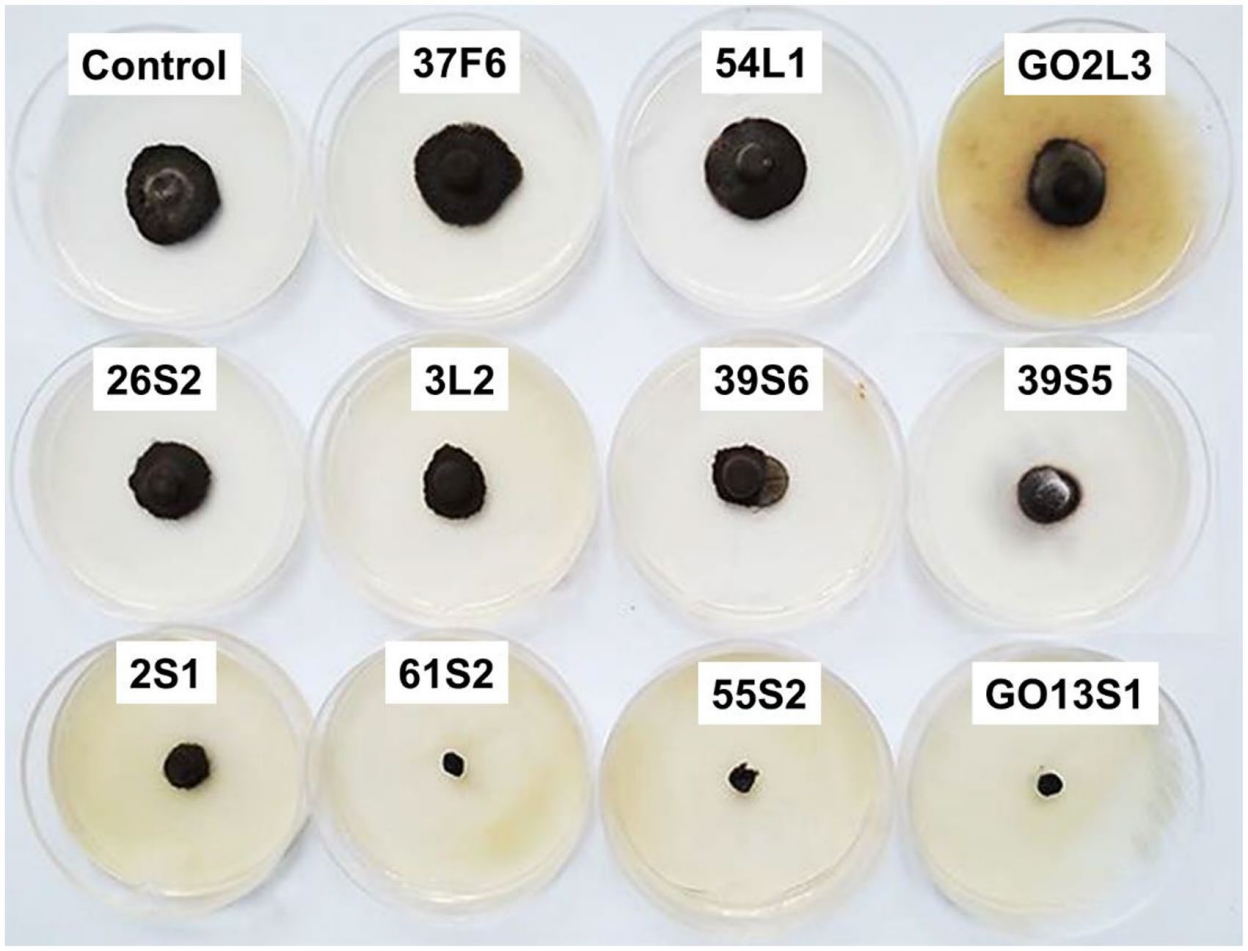
Effect of media-permeable metabolites of endophytic fungi isolated from apple on *Venturia inaequalis* mycelia growth in comparison to control after 1 month *in vitro*.

Figures 7, 8 display the results related to the media-permeable metabolites of 29 endophytic isolates on the mycelial growth of *V. inaequalis*. As shown, the metabolites of *Acremonium sclerotigenum* (Moreau & R. Moreau ex Valenta) W. Gams GO13S1, *Coniochaeta endophytica* A.H. Harrington & A.E. Arnold 55S2, and *Fusarium lateritium* Nees 61S2 lead to the maximum growth inhibition (100%) of *V. inaequalis*, followed by *Hydeomyces desertipleosporoides* Maharachch., H.A. Ariyaw., Wanas. & Al-Sadi GO8S2 (87.7%) and *Chaetomium globosum* Kunze 2S1 (78.7%).

**Figure 9:**
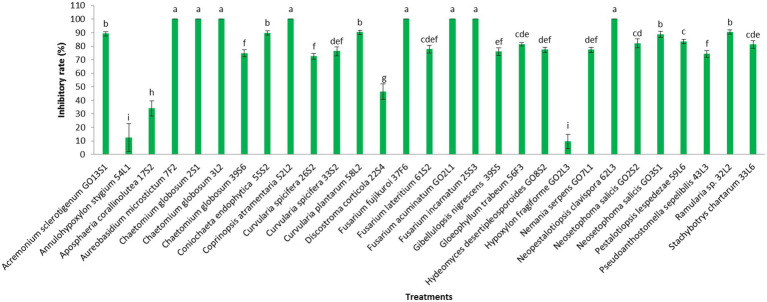
Effect of volatile organic compounds of endophytic fungi isolated from apple on *Venturia inaequalis* mycelia growth after 1 month *in vitro*. Data are the means ± SE of three replicates. Values of histograms with common letters are not significantly different at p ≤ 0.01 according to Duncan’s multiple range test.

**Figure 10:**
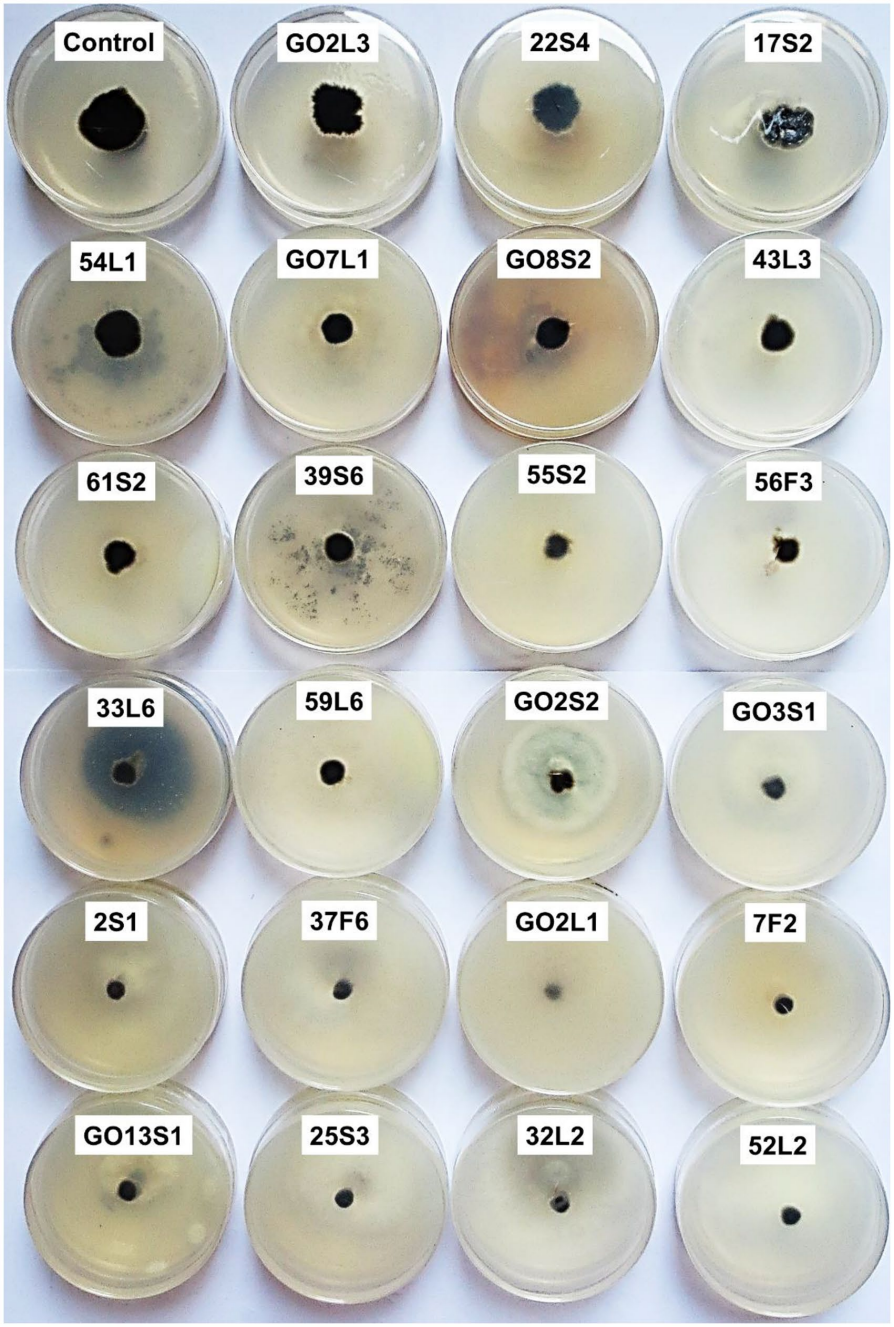
Effect of volatile organic compounds of endophytic fungi isolated from apple on *Venturia inaequalis* mycelia growth in comparison to control after 1 month *in vitro*.

Additionally, the VOC production was assessed in the isolates. The VOCs produced by the isolates of *Aureobasidium microstictum* (Bubák) W.B. Cooke 7F2, *Ch. globosum* 2S1, *Ch. globosum* 3 L2, *Coprinopsis atramentaria* 52 L2, *Fusarium fujikuroi* Nirenberg 37F6, *Fusarium acuminatum* Ellis & Everh. GO2L1, and *Fusarium incarnatum* (Desm.) Sacc. 25S3 completely prevented the mycelia growth of *V. inaequalis*. However, the minimum pathogen growth inhibition was detected in the VOCs of *Annulohypoxylon stygium* (Lév.) Y.M. Ju, J.D. Rogers & H.M. Hsieh 54 L1 (12.4%) and *Hypoxylon fragiforme* (Pers.) J. Kickx f. GO2L3 (9.6%). The VOCs of other isolates except *Aposphaeria corallinolutea* Gruyter, Aveskamp & Verkley 17S2 and *Discostroma corticola* (Fuckel) Brockmann 22S4 inhibited pathogen mycelia growth by more than 70% (Figures 9, 10).

### Phosphate solubilization and enzyme activity

Based on the results of the biological control tests, six isolates were selected for further evaluation, of which *Co. endophytica* 55S2 and *F. lateritium* 61S2 could solubilize inorganic phosphate with the ratio of 1.2 and 1.07, respectively (Table 3). All isolates except *Ch. globosum* 3 L2 represented cellulase activity, which the highest halo zone/colony diameter ratio (2.62) was recorded for *Au. microstictum* 7F2 (Table 3). Further, chitinase activity was observed in the isolates of *Ch. globosum* 2S1, *Ch. globosum* 3L2, and *F. lateritium* 61S2 with the halo zone/colony diameter ratio of 1.2, 1.2, and 1.1, respectively (Table 3).

**Table 3:** Phosphate solubilization and enzyme activity of selected isolates.

Endophytic fungi	Cellulase	Chitinase	Phosphate solubilization
*Acremonium sclerotigenum* GO13S1	1.96 ± 0.05	0.0 ± 0.00	0.0 ± 0.00
*Aureobasidium microstictum* 7F2	2.62 ± 0.13	0.0 ± 0.00	0.0 ± 0.00
*Chaetomium globosum* 2S1	1.03 ± 0.00	1.2 ± 0.05	0.0 ± 0.00
*Chaetomium globosum* 3 L2	0.00 ± 0.00	1.2 ± 0.00	0.0 ± 0.00
*Coniochaeta endophytica* 55S2	1.67 ± 0.03	0.0 ± 0.00	1.2 ± 0.05
*Fusarium lateritium* 61S2	1.25 ± 0.03	1.1 ± 0.04	1.07 ± 0.0

The means halo zone diameter/ colony diameter of three replications ± standard deviation is represented.

### Biocontrol assays under greenhouse conditions

**Figure 11:**
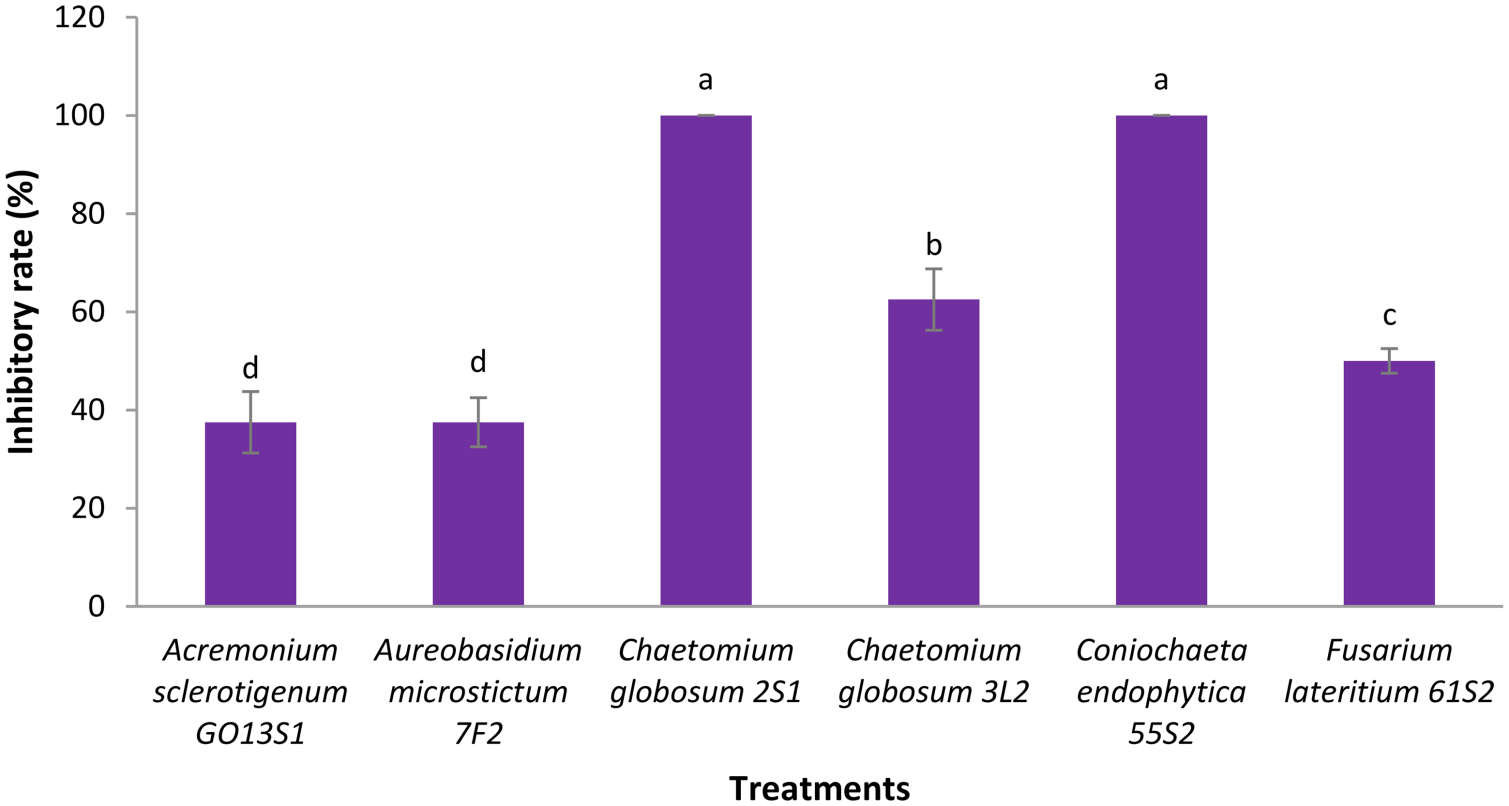
Antagonistic effect of endophytic fungi isolated from apple against apple scab disease on apple seedling after 1 month of pathogen inoculation under greenhouse conditions. Data are the means ± SE of three replicates. Values of histograms with common letters are not significantly different at p ≤ 0.01 according to Duncan’s multiple range test.

**Figure 12:**
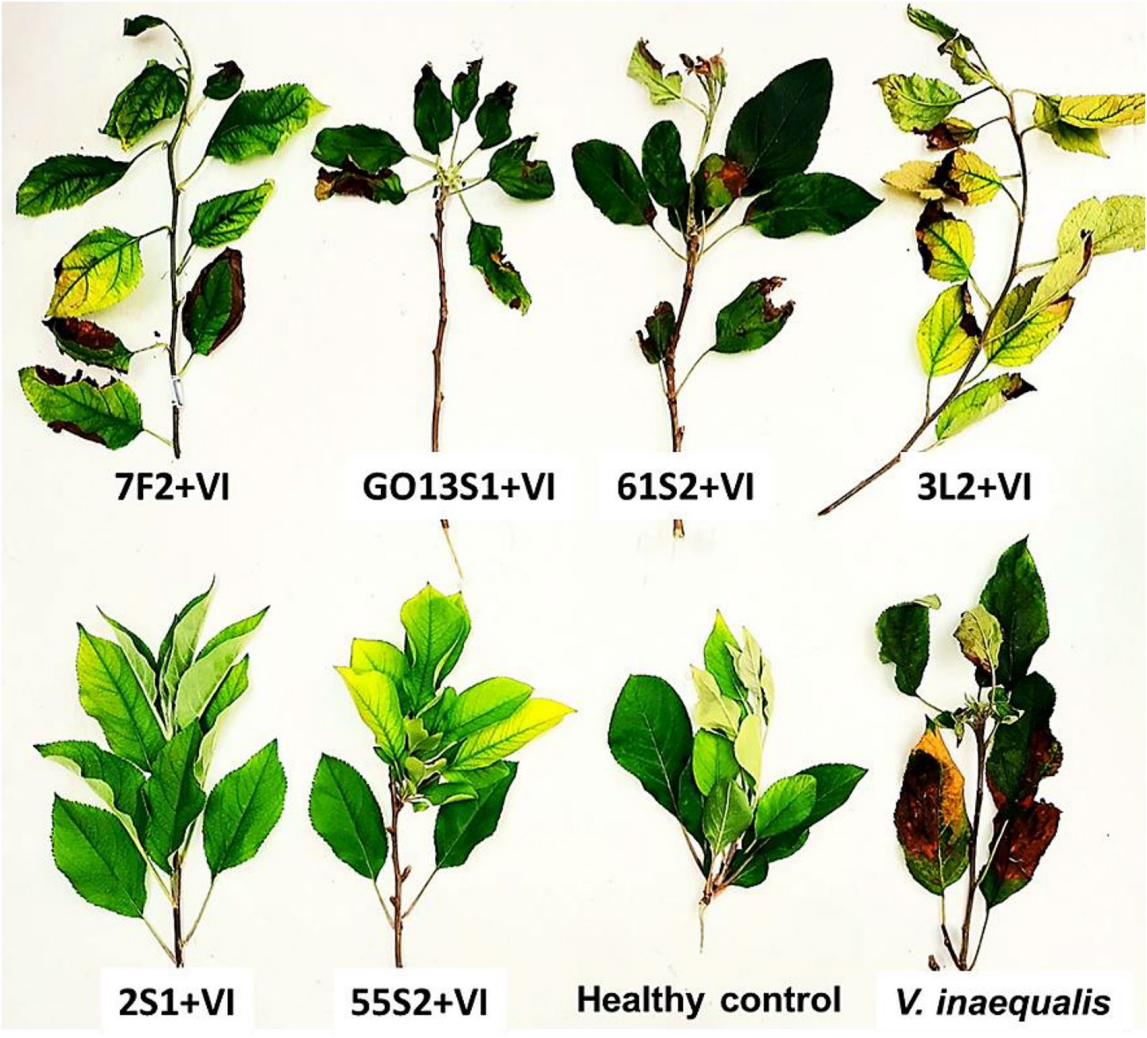
Effect of endophyte isolates on development of symptoms caused by *Venturia inaequalis* on apple leaves at 18–20°C after 1 month of pathogen inoculation in greenhouse. (Treatments: *Aureobasidium microstictum* 7F2, *Acremonium sclerotigenum* GO13S1, *Fusarium lateritium* 61S2, *Chaetomium globosum* 3 L2, *Chaetomium globosum* 2S1, *Coniochaeta endophytica* 55S2, VI: *Venturia inaequalis*).

As already mentioned, six isolates were further tested in whole-plant tests under greenhouse conditions. The results demonstrated a significant reduction in the apple scab severity after 1 month when endophytes were inoculated on the leaves 48 h before pathogen (Figures 11, 12). The *in vivo* tests revealed the complete control of apple scab disease by *Co. endophytica* 55S2 and *Ch. globosum* 2S1. Furthermore, the decrease in the apple scab severity was similar for *F. lateritium* 61S2 and *Ch. globosum* 3 L2, which ranged between 50 (*F. lateritium* 61S2) and 62% (*Ch. globosum* 3L2). Finally, *Au. microstictum* 7F2 and *Ac. sclerotigenum* GO13S1 declined the disease severity by 37.5% (Figures 11, 12).

## Discussion

The plant-associated habitat is considered as a dynamic environment, in which many factors affect the structure and composition of the species colonizing various tissues. The endophytic communities may spatially vary in many kinds of plants (Rivera-Orduña et al., 2011). In addition, microorganism population can be different in natural forest and agroecosystem due to the use of synthetic chemicals by farmers. The present study highlighted the endophytic fungi associated with the wild and Iranian endemic apple cultivars which are mostly spread along the Caspian Sea coast in the north of Iran. The results revealed the differences in the apple branch, fruit, and leaf tissues in terms of the richness and abundance of endophytic fungi. Among the 417 endophytic isolates under study, 243, 112, and 62 were obtained from leaves, branches, and fruits, respectively. Generally, leaves carry more endophytic fungi than the stem due to the exposure of larger surface area to the outer environment and presence of numerous stomata which facilitate the entry of fungal hyphae (Gond et al., 2012). Camatti-Sartori et al. (2005) reported the greatest total number of endophytic isolates from the orchards under organic cultivation compared to the integrated and conventional cultivation systems. Alijani et al. (2016a) separated 14.37, 28.34, and 57.28% of endophytic fungal isolates from the apple leaf, annual and biennial branches, and bark in the commercial orchards of West Azerbaijan province, Iran (n: 350). These results are inconsistent with those of the present study regarding endophytic fungal diversity on the wild and endemic apple cultivars from the forests and natural regions. Alijani et al. (2016a) suggested the possible effect of fungicide application in commercial orchard on the endophytic fungal abundance of apple leaves. However, many other factors such as climate, orchard location, and host cultivar may influence the abundance and diversity of endophytic fungi. Afandhi et al. (2018) found that the abundance and diversity of endophytic fungi were maximized in mature apple leaves compared to the young and old ones. According to Arrigoni et al. (2020), bark age and orchard location strongly affected fungal and bacterial diversity. Further, scab disease management had an effect on the abundance of some taxa depending on bark age, orchard location, and sampling time (Arrigoni et al., 2020). Interestingly, Olivieri et al. (2021) mentioned the presence of significant differences between canker-resistant and susceptible apple cultivars with respect to the endophyte community.

In the present study, 417 endophytic fungi were separated from apple, which mostly belonged to Ascomycota (77.93%). The genera *Alternaria*, *Cladosporium*, *Nigrospora*, *Colletotrichum*, *Fusarium*, *Chaetomium*, and *Curvularia* were the most frequent fungi, the isolate number of which was 50, 49, 39, 23, 16, 9, and 8, respectively. Camatti-Sartori et al. (2005) identified genera *Colletotrichum*, *Xylaria*, and *Botryosphaeria* as the most common endophytic fungi of apple in Brazil. Based on the results of Liu et al. (2018), Ascomycota (47.8%), Mucoromycota (31.1%), and Basidiomycota (11.6%) were the dominant endophytic fungal phyla in all apple samples studied in the USA. In general, *Zoophthora* Bałazy & Manole (31%), *Cladosporium* (17.3%), and *Aureobasidium* (11%) constituted more than 59% of the detected fungi, and the next genera were *Alternaria* (5.6%) and *Aspergillus* (1.6%), respectively (Liu et al., 2018). Alijani et al. (2016a,b) reported the presence of 12 genera of endophytic fungi, *Alternaria*, *Arthrinium*, *Aspergillus*, *Chaetomium*, *Cytospora* Ehrenb., *Dicyma*, *Doratomyces*, *Paraconiothyrium*, *Periconia* Tode, *Stemphylium*, *Trichoderma*, and *Trichothecium*, on the apple cultivated in the West Azerbaijan province, Iran. Muresan (2017) gathered a collection of 60 fungal isolates, mostly obtained from apple tree roots (78%) in Canada, 60% of which were *Penicillium* or *Trichoderma* species. Liu et al. (2017) referred to *Epicoccum*, *Chaetomium*, *Biscogniauxia*, *Neoseptophoma*, and *Penicillium* as the most species identified in the culturable endophytes from apple shoots in New Zealand. According to Afandhi et al. (2018), *Aspergillus* was dominant in the endophytic fungal isolates of apple.

The biological control of plant pathogens instead of synthetic fungicides adequately protects plants, humans and other animals, and natural environment. The endophytic fungi play a key role in plant protection against biotic and abiotic stresses (Lugtenberg et al., 2016) through the various modes of action such as the mycoparasitism, antibiosis, competition, and plant resistance induction (Latz et al., 2018). The mechanisms may act coordinately, and their importance in the biological control process depends on the antagonist strain, pathogenic fungus, host plant, and environmental conditions (Golafrouz et al., 2020).

Many active metabolites are produced by antagonistic fungi, some of which possess significant biological activities like cytotoxicity, enzyme inhibition, and antibiosis (Yang S. X. et al., 2011; Yang S. Z. et al., 2011). Furthermore, some antagonistic fungi secrete various hydrolytic enzymes such as chitinase, glucanase, and protease to effectively digest the cell walls of their competitors, inhibit their ss growth, or surpass their capacity for metabolizing available resources leading to competitive exclusion in substrate (Wilkins et al., 2003). The endophytic fungi colonize plant tissues, as well as obtaining nutrients and highly-active cellulases produced to help the host plant defend themselves against invading pathogens (Marques et al., 2018). Therefore, some enzyme activities of endophytes such as cellulase are important in biological control. Additionally, phosphorus is known as a major plant nutrient, the insoluble calcium phosphate salts of which are mostly formed in the presence of a high concentration of calcium ions (Chen and Barak, 1982). Thus, the ability of endophytes to solubilize inorganic phosphate can help plant by improving their growth. In the present study, the six isolates of *Ac. sclerotigenum* GO13S1, *Co. endophytica* 55S2, *F. lateritium* 61S2, *Au. microstictum* 7F2, *Ch. globosum* 2S1, and *Ch. globosum* 3 L2 were selected for greenhouse tests based on the results of media-permeable metabolites and VOC tests. Among the intended isolates, *Co. endophytica* 55S2 and *F. lateritium* 61S2 had the ability to solubilize inorganic phosphate, which the ability should be investigated in soil environment to affects plant growth and disease biocontrol. The cellulase activity was observed in all isolates except *Ch. globosum* 3 L2, while *Ch. globosum* 2S1, *Ch. globosum* 3 L2, and *F. lateritium* 61S2 exhibited chitinase activity. The results demonstrated the successful biological control of apple scab disease following the use of endophytic fungi in greenhouse so that *Co. endophytica* 55S2 and *Ch. globosum* 2S1 completely controlled the disease on apple seedling leaves.

The various studies around the world have revealed the biological control potential of different apple endophytic fungi against apple fungal pathogens. For example, Alijani et al. (2016b) evaluated the antifungal potential of 15 endophytic species of apple against *D. bulgarica in vitro*. They found that pathogen mycelia growth significantly reduced by *Trichoderma harzianum* Rifai and *Trichoderma longibrachiatum* Rifai. According to Muresan (2017), 55 endophytic isolates of apple among 60 ones significantly prevented *V. inaequalis* growth *in vitro* and *F. oxysporum* FRS09 was determined as the most effective isolate with 83% inhibition. Doolotkeldieva and Bobusheva (2017) examined the two biological control agents of *Trichoderma viride* Pers. and *Streptomyces* sр. against apple scab disease *in vitro* and field conditions. The application of *T. viride* within 35 days completely stopped the disease in seedling leaves, while *Streptomyces* sp. isolates were less effective than the *T. viride*. Further, there was a diverse range of potential biological control agents for organic apple production. Liu et al. (2017) reported the potential of the endophytes isolated from apple tissues (i.e., *Epicoccum*, *Chaetomium*, *Biscogniauxia*, *Neoseptophoma*, and *Penicillium*) for using in the sustainable control of *N. ditissima*. *Chaetomium globosum* is one of the most common species utilized in biological control against various phytopathogens such as *V. inaequalis* (Soytong and Ratanacherdchai, 2005; Zhang et al., 2010). The results of thin-layer chromatography studies reflected the production of different secondary metabolites such as chetomin, BHT, mollicelin G along with chaetoglobosin by this species (Biswas et al., 2012). Several *Chaetomium* spp. such as *Ch. cupreum* L.M. Ames and *Ch. globosum* produce resistance-inducing substances which prevent many plant diseases like *Pythium aphanidermatum* (Edson) Fitzp. in sugarcane, wilt symptoms in grain seedlings, and apple scab incited by *Venturia* spp., as well as decreasing tomato Fusarium wilt, and inhibiting the growth of pathogenic *Rhizoctonia solani* J.G. Kühn and *Botrytis* spp. (Soytong and Ratanacherdchai, 2005).

## Conclusion

In this study, we have identified *Co. endophytica* 55S2, and *Ch. globosum* 2S1 as the most potent endophytic fungal isolates for controlling apple scab disease caused by *V. inaequalis* under greenhouse conditions. These isolates could therefore be considered the best candidates for development of endophytic-based biofungicide and could be integrated as a component in a sustainable integrated apple management strategy for scab. However, further studies are warranted to clearly understand the underlying mechanisms by which the presence of endophytic fungi affect *V. inaequalis* as well as validate the findings under field conditions on different cultivars of apple.

## Data availability statement

The data presented in the study are deposited in the GenBank repository, accession numbers presented in Table 1.

## Author contributions

LE designed and directed the research and wrote the manuscript. HE gave advice during experiments. LE and SHR carried out experiments. All authors contributed to the interpretation of the results. All authors contributed to the article and approved the submitted version.

## Funding

This work was supported by the Iran National Science Foundation (INSF) (Grant No. 97021003), and University of Tehran, Iran.

## Conflict of interest

The authors declare that the research was conducted in the absence of any commercial or financial relationships that could be construed as a potential conflict of interest.

## Publisher’s note

All claims expressed in this article are solely those of the authors and do not necessarily represent those of their affiliated organizations, or those of the publisher, the editors and the reviewers. Any product that may be evaluated in this article, or claim that may be made by its manufacturer, is not guaranteed or endorsed by the publisher.
