# Supplementary material for: Apple Endophytic fungi and their antagonism against apple scab disease
Source: Front Microbiol. 2022 Nov 7;13:1024001. doi: 10.3389/fmicb.2022.1024001 (PMC9677113; doi:10.3389/fmicb.2022.1024001)
Supplement: Supplementary file 1 [file Table_1.DOCX]

Supplementary Material

**Supplementary TABLE S1** GenBank accession numbers of the sequences used in the phylogenetic analysis*.*

| Species | Culture accession number (s) | Source | Origin | GeneBank accession numbers | | |
| --- | --- | --- | --- | --- | --- | --- |
|  |  |  |  | *gapdh* | *tef-1α* | ITS |
| *Curvularia buchloes* | CBS 246.49 | *Buchloe dactyloides* | USA | KM061789 |  |  |
| *Curvularia ellisii* | CBS 193.62 | culture from holotype | - | LT715811 |  |  |
|  | CBS 127083 | *Dactyloctenium aegyptium* | Australia | MN688832 |  |  |
| *Curvularia hominis* | **IRAN 4400C** | ***Malus orientalis*** | **Iran** | **MZ339272** |  |  |
|  | HNWB120 | *Zea mays* | China | KX100868 |  |  |
|  | UTHSC 08-849 | Clinical sample | - | HF565483 |  |  |
| *Curvularia muehlenbeckiae* | UTHSC 08-2905 | *-* | - | LT715807 |  |  |
| *Curvularia pisi* | CBS 190.48 | *Pisum sativum* | Canada | KY905690 |  |  |
| *Curvularia rouhanii* | CBS 144674 | *Syngonium vellozianum* | Iran | MG428694 |  |  |
|  | CBS 144675 | *Eucalyptus* sp. | Iran | MG428696 |  |  |
| *Curvularia spicifera* | **IRAN 4370C** | ***Malus domestica*** | **Iran** | **MZ339270** |  |  |
|  | **IRAN 4371C** | ***Malus domestica*** | **Iran** | **MZ339271** |  |  |
|  | CBS 198.31 | *-* | Switzerland | LT715814 |  |  |
|  | BC21ss2 | *Buchloe dactyloides* | USA | KC928091 |  |  |
| *Curvularia tribuli* | CBS 126975 | *Tribulus terrestris* | South Africa | MN688852 |  |  |
| *Curvularia variabilis* | CPC 28813 | *Digitaria ciliaris* | Thailand | MF490842 |  |  |
|  | CPC 28816 | *Imperata cylindrica* | Thailand | MF490845 |  |  |
| *Alternaria tenuissima* | IRAN 2428C | Quince | Iran | MN160228 |  |  |
| *Fusarium acuminatum* | **IRAN 4374C** | ***Malus domestica*** | **Iran** |  | **MZ339269** |  |
|  | F6 | - | Serbia |  | MH595499 |  |
|  | LD1508081502 | *Orobanche coerulescens* | China |  | MF523228 |  |
| *Fusarium avenaceum* | M247 | *Solanum tuberosum* | Poland |  | KP400697 |  |
|  | Z172B | *Solanum tuberosum* | Poland |  | KP674208 |  |
| *Fusarium camptoceras* | CBS 193.65 | - | Japan |  | AB820706 |  |
|  | NRRL 13381 | - | - |  | GQ915504 |  |
| *Fusarium fujikuroi* | **IRAN 4373C** | ***Malus domestica*** | **Iran** |  | **MZ339267** |  |
|  | EF46 | maize | Spain |  | MN861769 |  |
|  | EF54 | maize | Spain |  | MN861772 |  |
| *Fusarium globosum* | CBS 430.97 | *Zea mays* seed | South Africa |  | LT746231 |  |
|  | CBS:428.97 | - | - |  | MT010993 |  |
| *Fusarium lateritium* | **IRAN 4470C** | ***Malus domestica*** | **Iran** |  | **MZ339268** |  |
|  | F156N1 | aerobiological sampling | USA |  | KC874765 |  |
|  | F157N3 | aerobiological sampling | USA |  | KC874804 |  |
| *Fusarium langsethiae* | NRRL 34176 | *Spartina alterniflora* | Atlantic salt |  | GQ856980 |  |
| *Fusarium incarnatum* | **IRAN 4372C** | ***Malus domestica*** | **Iran** |  | **MZ339266** |  |
|  | Lb2 | *Oryza sativa* | Spain |  | JF715935 |  |
|  | ZJ11 | rice | China |  | MT560647 |  |
| *Fusarium torreyae* | CBS 133858 | *Torreya taxifolia* | USA |  | MW928845 |  |
| *Fusarium venenatum* | Z335A | *Solanum tuberosum* | Poland |  | KP400712 |  |
|  | Ch61I | *Solanum tuberosum* | Poland |  | KP400742 |  |
| *Nectria illudens* | NRRL 22090 | - | USA |  | AF178326 |  |
| *Acremonium sclerotigenum* | **IRAN 4360C** | ***Malus domestica*** | **Iran** |  |  | **MZ151394** |
|  | 3H6 | **-** | Mexico |  |  | MK725873 |
| *Alternaria infectoria* | **IRAN 4380C** | ***Malus orientalis*** | **Iran** |  |  | **MZ151363** |
|  | CNRMA10.1102 | - | France |  |  | KP131537 |
| *Alternaria tenuissima* | **IRAN 4362C** | ***Malus domestica*** | **Iran** |  |  | **MZ151368** |
|  | GXBJ-1 | - | China |  |  | MN893910 |
| *Aposphaeria corallinolutea* | **IRAN 4381C** | ***Malus domestica*** | **Iran** |  |  | **MZ151364** |
|  | MFLU:16-2412 | - | Russia |  |  | MT177916 |
| *Annulohypoxylon stygium* | **IRAN 4393C** | ***Malus domestica*** | **Iran** |  |  | **MZ151378** |
|  | 300 | *Cuscuta campestris* | Iran |  |  | KP170485 |
| *Anthostomella eucalyptorum* | CBS 120036 | *Eucalyptus* sp. | Indonesia |  |  | DQ890026 |
|  | 2741 | *Elymus farctus* | Spain |  |  | AM922205 |
| *Aspergillus terreus* | **IRAN 4361C** | ***Malus domestica*** | **Iran** |  |  | **MZ151366** |
|  | CF3 | *Phaseolus vulgaris* | Egypt |  |  | LC508264 |
| *Aspergillus versicolor* | **IRAN 4365C** | ***Malus orientalis*** | **Iran** |  |  | **MZ151374** |
|  | R47 | Barber shops | Kenya |  |  | MT420642 |
| *Aureobasidium microstictum* | **IRAN 4378C** | ***Malus orientalis*** | **Iran** |  |  | **MZ151360** |
|  | CBS 342.66 | - | Germany |  |  | EU167608 |
| *Chaetomium globosum* | **IRAN 4355C** | ***Malus domestica*** | **Iran** |  |  | **MZ151358** |
|  | **IRAN 4356C** | ***Malus domestica*** | **Iran** |  |  | **MZ151359** |
|  | **IRAN 4364C** | ***Malus domestica*** | **Iran** |  |  | **MZ151371** |
|  | B1/2/VF | book collections | Poland |  |  | KX901285 |
|  | C-56 | **-** | India |  |  | KT357693 |
| *Colletotrichum fructicola* | **IRAN 4395C** | ***Malus orientalis*** | **Iran** |  |  | **MZ151382** |
|  | SXY-2 | *Wurfbainia villosa* | China |  |  | MN784184 |
| *Colletotrichum gloeosporioides* | **UT53L1** | ***Malus domestica*** | **Iran** |  |  | **MZ151386** |
|  | SR1 | *Huperzia serrata* | China |  |  | KP689241 |
| *Colletotrichum godetiae* | **IRAN 4391C** | ***Malus orientalis*** | **Iran** |  |  | **MZ151375** |
|  | F8231 | *Chloranthus japonicas* | China |  |  | MN429277 |
|  | Acf-14 | **-** | China |  |  | MK795225 |
| *Coniochaeta cymbiformispora* | NBRC 32199 | swamp soil | Japan |  |  | LC146726 |
| *Coniochaeta cephalothecoides* | L821 | *Trametes cinnabarina* | China |  |  | KY064029 |
|  | TPYD-10 | - | China |  |  | MN544932 |
| *Coniochaeta endophytica* | **IRAN 4366C** | ***Malus domestica*** | **Iran** |  |  | **MZ151379** |
|  | AEA 9094 | *Platycladus orientalis* | USA |  |  | EF420005 |
|  | AEA 9055 | *Platycladus orientalis* | USA |  |  | MK614056 |
| *Coniochaeta ligniaria* | TP131 | *Cremastra appendiculata* | China |  |  | MT920581 |
| *Coprinopsis atramentaria* | **IRAN 4392C** | ***Malus domestica*** | **Iran** |  |  | **MZ151377** |
|  | EF-373 | **-** | China |  |  | MG132086 |
|  | xsd08072 | **-** | China |  |  | FJ478115 |
| *Discostroma corticola* | **IRAN 4387C** | ***Malus domestica*** | **Iran** |  |  | **MZ151365** |
|  | ER2097 | apple | Italy |  |  | MF069255 |
| *Gibellulopsis nigrescens* | **IRAN 4389C** | ***Malus domestica*** | **Iran** |  |  | **MZ151370** |
|  | CCJ1(1) | tobacco | China |  |  | KM268699 |
| *Gleoephyllum trabeum* | **IRAN 4367C** | ***Malus domestica*** | **Iran** |  |  | **MZ151380** |
|  | CFMR:Mad-617-R | **-** | USA |  |  | KJ995949 |
|  | CFMR:Boat-194 | **-** | **-** |  |  | KJ995922 |
| *Hydeomyces desertipleosporoides* | **IRAN 4386C** | ***Malus domestica*** | **Iran** |  |  | **MZ151393** |
|  | SQUH 101 | - | Oman |  |  | NR_164295 |
| *Hypoxylon fragiforme* | **IRAN 4382C** | ***Malus domestica*** | **Iran** |  |  | **MZ151387** |
|  | BO390 | - | Germany |  |  | EF155523 |
|  | ARSL_071114.22 | *Vitis riparia* | Canada |  |  | KX869947 |
| *Nemania serpens* | **IRAN 4385C** | ***Malus domestica*** | **Iran** |  |  | **MZ151392** |
|  | SER44_E.2 | *Castanea sativa* | Portugal |  |  | MN996233 |
| *Neopestalotiopsis clavispora* | **IRAN 4369C** | ***Malus domestica*** | **Iran** |  |  | **MZ151385** |
|  | Y-6 | Crinoidea | China |  |  | KP281446 |
|  | 4 | *Mangifera indica* | Italy |  |  | JX875595 |
| *Neoscytalidium dimidiatum* | **IRAN 4363C** | ***Malus domestica*** | **Iran** |  |  | **MZ151369** |
|  | URF_Pt01 | potato | Turkey |  |  | MT010216 |
| *Neosetophoma salicis* | **IRAN 4384C** | ***Malus domestica*** | **Iran** |  |  | **MZ151389** |
| *Neosetophoma salicis* | **IRAN 4383C** | ***Malus domestica*** | **Iran** |  |  | **MZ151388** |
|  | MFLU 17-0118 | *Salix* sp*.* | Uzbekistan |  |  | MK608025 |
| *Neosetophoma* *clematidis* | MFLUCC 13-0734 | culture from holotype | *-* |  |  | NR_154228 |
| *Neosetophoma* *lunariae* | CPC 26671 | *Lunaria annua* | Germany |  |  | NR_154242 |
| *Neosetophoma* *shoemakeri* | MFLUCC 17-0780 | *Malva* sp*.* | United Kingdom |  |  | MG844346 |
| *Nigrospora oryzae* | **IRAN 4358C** | ***Malus domestica*** | **Iran** |  |  | **MZ151390** |
|  | **IRAN 4399C** | ***Malus domestica*** | **Iran** |  |  | **MZ151376** |
|  | **IRAN 4398C** | ***Malus domestica*** | **Iran** |  |  | **MZ151373** |
|  | **IRAN 4394C** | ***Malus domestica*** | **Iran** |  |  | **MZ151384** |
|  | CR34 | *Caulerpa racemosa* | India |  |  | MH748173 |
|  | 10LW-2 | *Citrus sinensis* | Iran |  |  | KU375674 |
| *Paecillomyces maximus* | **IRAN 4368C** | ***Malus orientalis*** | **Iran** |  |  | **MZ151383** |
|  | 3 | beef | North Africa |  |  | MH345843 |
| *Penicillium chrysogenum* | **IRAN 4357C** | ***Malus orientalis*** | **Iran** |  |  | **MZ151362** |
|  | EG 13-1 | ancient tombs | Egypt |  |  | MT730080 |
| *Pestalotiopsis lespedezae* | **IRAN 4396C** | ***Malus orientalis*** | **Iran** |  |  | **MZ151381** |
|  | JL9 | *Huperzia serrata* | China |  |  | KM513577 |
|  | EC12A | - | China |  |  | EF055200 |
| *Pestalotiopsis diploclisia* | CBS 115587 | *Diploclisia glaucescens* | China |  |  | KM199320 |
| *Pestalotiopsis papuana* | CBS 887.96 | *Cocos nucifera* | Papua New Guinea |  |  | KM199318 |
| *Pseudoanthostomella sepelibilis* | **IRAN 4390C** | ***Malus domestica*** | **Iran** |  |  | **MZ151372** |
|  | F-160 | *-* | Spain |  |  | AY908989 |
| *Ramularia* sp. | **IRAN 4388C** | ***Malus orientalis*** | **Iran** |  |  | **MZ151367** |
|  | CBS:124861 | **-** | Netherland |  |  | KP894302 |
| *Ramularia endophylla* | FeC150 | *Fraxinus excelsior* | Poland |  |  | MW446998 |
| *Stachybotrys chartarum* | **IRAN 4397C** | ***Malus domestica*** | **Iran** |  |  | **OM674386** |
|  | CH | grapevine | USA |  |  | KR909154 |
|  | 93A | air | Colombia |  |  | KP067272 |
| *Talaromyces verruculosus* | **IRAN 4359C** | ***Malus domestica*** | **Iran** |  |  | **MZ151391** |
|  | MR39-1 | *Myricaria laxiflora* | China |  |  | KU324796 |
| *Venturia inaequalis* | **IRAN 4379C** | ***Malus orientalis*** | **Iran** |  |  | **MZ151361** |
|  | XJTKSPG5-4 | - | China |  |  | MN958673 |

The surveyed isolates in the current study are indicated in bold.
